# Structural Insights into Substrate Recognition and Processing by the 20S Proteasome

**DOI:** 10.3390/biom11020148

**Published:** 2021-01-24

**Authors:** Indrajit Sahu, Michael H. Glickman

**Affiliations:** Faculty of Biology, Technion-Israel Institute of Technology, 32000 Haifa, Israel

**Keywords:** 20S proteasome, protein degradation, intrinsically disordered proteins, enzyme functional cycle, peptides, peptidome, proteome, oxidative stress

## Abstract

Four decades of proteasome research have yielded extensive information on ubiquitin-dependent proteolysis. The archetype of proteasomes is a 20S barrel-shaped complex that does not rely on ubiquitin as a degradation signal but can degrade substrates with a considerable unstructured stretch. Since roughly half of all proteasomes in most eukaryotic cells are free 20S complexes, ubiquitin-independent protein degradation may coexist with ubiquitin-dependent degradation by the highly regulated 26S proteasome. This article reviews recent advances in our understanding of the biochemical and structural features that underlie the proteolytic mechanism of 20S proteasomes. The two outer α-rings of 20S proteasomes provide a number of potential docking sites for loosely folded polypeptides. The binding of a substrate can induce asymmetric conformational changes, trigger gate opening, and initiate its own degradation through a protease-driven translocation mechanism. Consequently, the substrate translocates through two additional narrow apertures augmented by the β-catalytic active sites. The overall pulling force through the two annuli results in a protease-like unfolding of the substrate and subsequent proteolysis in the catalytic chamber. Although both proteasomes contain identical β-catalytic active sites, the differential translocation mechanisms yield distinct peptide products. Nonoverlapping substrate repertoires and product outcomes rationalize cohabitation of both proteasome complexes in cells.

## 1. Introduction

Two major species of proteasome coexist in most cells: the 20S proteasome as a standalone complex, and the 20S complex as a core particle that is associated with regulatory particles or activators [1,2,3]. The 20S is a self-compartmentalized protease complex [4] that carefully selects substrates having substantiate disordered or misfolded stretches [5,6,7] and proteolyses them once they enter into the inner chamber. Under in vitro conditions without any activators or associated unfoldase activities, the 20S proteasome proteolyses unstructured proteins or extended polypeptides [8,9,10,11,12,13,14]. Interestingly, archaea and some bacteria have 20S proteasomes alongside other ATP-dependent proteases, which supports the idea of the 20S complex being a primordial protein-degrading machine [15]. Usually, these simple 20S proteasomes are made up of 14 copies of α and β subunits each [16,17], intermittently aided by a loosely associated homomeric ring of ATPases [15,18,19]. All β subunits in archaeal proteasomes are catalytically active proteases arranged in two concerted rings around an enclosed catalytic chamber, with the 14 α subunits forming two outer rings through which substrate enters. In contrast to archaea, 20S proteasomes in eukaryotes display greater complexity, with seven different types of α and β subunits each, stacked in a four-ringed 20S complex. The symmetry mismatch between seven different α subunits in each outer ring enables a gating mechanism, which provides a regulatory system for substrate entry [20,21,22,23]. Regarding the β subunits, out of seven only three retain proteolytic activity in eukaryotes influencing the peptide product outcome [24].

**Figure 1:**
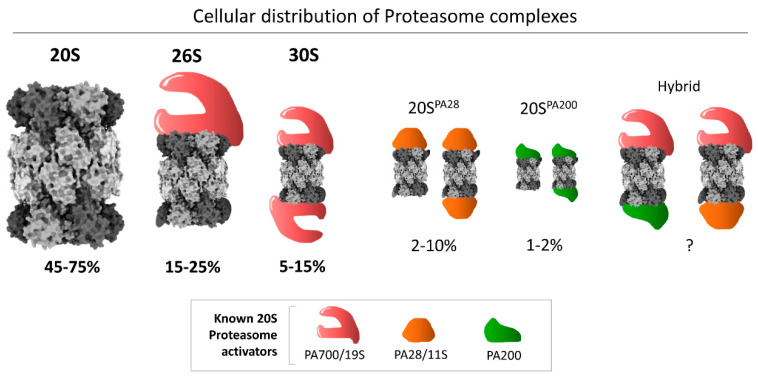
Cellular distribution of different proteasome complexes in mammalian cells. The 20S model figure was generated by ChimeraX using the 20S cryo-EM structure [49]. The average percentages are calculated based on published reports [3]. The size of each proteasome species corresponds to the average value of the range denoted to visually illustrate their relative abundance in cell lines that have been quantified.

In eukaryotic cells, 20S proteasomes function independently and act directly on disordered proteins [25,26,27,28,29] or oxidized proteins [6,14,26,30,31], substantiating its potential role as a stand-alone protease. Nevertheless, its activity is augmented by various activators that attach to the 20S proteasome, aiding substrate recruitment and processing, thereby broadening its substrate repertoire. The 19S regulatory particle (RP) is the major activator that enables the 20S proteasome to degrade virtually any protein tagged with the small protein modifier ubiquitin [32,33]. Either one or two 19S RPs can attach to a single 20S catalytic core particle (CP) to form the singly capped 26S, or the doubly capped 30S, respectively (Figure 1) [34]. We refer readers to a number of recent reviews on 26S proteasomes [35,36,37,38,39,40]. Additional, simpler, non-ATPase activators can also associate with 20S CPs (e.g., PA200, PA28; [3,18,41,42,43,44,45,46,47,48]), although their mode of action in substrate degradation is vague and will not be detailed in this review.

Although the 20S complex as a core particle is an integral part of all species of larger complex proteasomes in eukaryotes [10,50], it is quite abundant as a free complex in many cell types, estimated in some studies at ~50% of all proteasome species [3] (Figure 1). It has been suggested that free 20S complexes may be proteasome assembly intermediates, 26S breakdown products (due to disassembly), or stand-alone proteolytic enzymes [1,5,6,7,14,21,51,52,53,54,55,56,57]. Since the ratio of 20S to 26S proteasome varies across different cellular conditions, a dynamic equilibrium between the two species may be part of an adaptive response to cellular needs [58]. Although its functionality as an independently active enzyme under cellular conditions is a matter of debate, recent advances have highlighted a role for 20S as a functional proteasome in cells. Various reports suggest a role of 20S as an emergency proteasome under cellular stress, for example oxidative stress or hypoxic stress [25,49,56,59,60,61], to provide survival benefits under proteotoxicity [26]. 

Any physiological condition that demands an alteration to the proteome or impairs protein function requires enhanced capacity to remove the unnecessary load. Common stress conditions such as oxidation, temperature, ionization or toxins directly damage proteins but also inevitably affect the ubiquitin–proteasome machinery. Indeed these stress conditions can lead to 26S accumulation in storage granules [62,63,64], its disassembly [1,2,65], ubiquitination [66], or proteophagy [67,68,69]. Nevertheless, 20S CPs are relatively resistant to oxidation damage compared to 26S proteasomes and persist as a stable complex under such conditions [57,70]. Fine-tuning the proteolytic machinery by altering the proteasome species ratio may be a strategy which cells utilize to survive under proteotocixity. For instance, ischemic-related hypoxia, a pathological condition of heart failure, is characterized by oxidative stress [71], and disassembly of the 26S proteasome [72,73]. While many studies have focused specifically on the decline of the 26S proteasome in acute conditions such in heart failure and during aging in general, residual 20S proteasome activity may contribute to the removal of misfolded or damaged proteins under a variety of stress conditions. Hence, it is most likely that 20S proteasomes play a role under stress conditions, yet how they serve to alleviate proteotoxicity is unclear. We refer readers to a number of recent reviews that highlight the participation of the 20S proteasomes in biological regulation or in the potential substrate repertoire [5,7,74,75].

**Figure 2:**
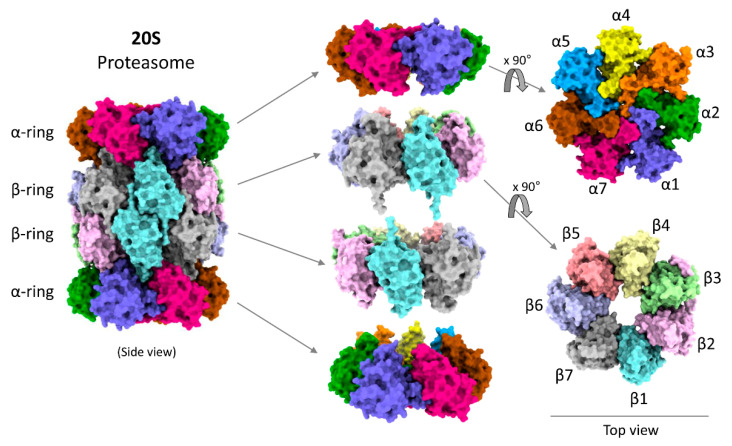
Model structure of human 20S proteasome. Model figures were generated by ChimeraX using the 20S cryo-EM structure [49].

Several structural studies on 20S proteasomes by crystallography, cryo-EM or atomic force microscopy have detailed the arrangements of all the 28 subunits to their atomic resolution (Figure 2) [76] and modes of substrate association [77,78,79]. Although 20S proteasomes can degrade proteins, details of their functional cycle and associated conformational changes are still obscure. In this review we correlate the structural information with biochemical knowledge to describe how the structural features of 20S proteasomes are competent to recognize and degrade their preferred substrates. The following review will focus on the aspects of 20S proteasome structure–function correlation based on recent advancement of knowledge and provide predictive models for its degradation functional cycle. 

## 2. Substrate Degradation Signals for 20S Proteasomes

Preferred substrates for degradation by 20S proteasomes are unstructured, unfolded or misfolded proteins. Various studies have addressed the ability of 20S proteasomes to degrade proteins of this category both in in vitro and in vivo conditions [9,25,26,28,49,59,80,81,82,83]. Hence, the targeting signal to 20S proteasomes is considered to be an unstructured polypeptide segment on a substrate. Many proteins possess intrinsic unstructured segments, but often the segments are either concealed within the protein itself [84], or protected/stabilized by binding partners or chaperones [85]. Once exposed, the segment could target the entire protein for degradation. It has been well reported that upon oxidation various proteins (e.g., α-Lactalbumin) become substrates of the 20S proteasome under in vitro conditions [78]. Under hypoxia or oxidative stress conditions oxidized cellular proteins are readily degraded by 20S proteasomes [61]. Since oxidative damage can lead to increase disorderedness and decrease protein stability, it could affect the structure of proteins by exposing the buried or intrinsic unstructured segments that might act as signals for 20S proteasome recognition and proteolysis. Furthermore, 20S proteasomal S-glutathionylation is a relevant adaptive response to oxidative stress that is capable of sensing the intracellular redox environment, leading to the removal of oxidized proteins via a process that is not dependent upon ubiquitylation and ATP consumption [86,87]. Hence, cellular oxidative conditions regulate 20S proteasome activity and specificity by modifying both proteins and proteasomes.

Proteins with a substantial measure of disorderedness have been named intrinsically disordered proteins (IDPs), and a role for 20S proteasomes in degrading them has been proposed [59]. Nevertheless, a short unstructured segment does not imply that the 20S proteasome is the primary protease for their removal, as most IDPs also undergo ubiquitination which leads to degradation by 26S proteasomes [88]. Since ubiquitinated substrates are preferentially degraded by 26S proteasomes, it is reasonable to assume that the non-ubiquitinated IDPs would be degraded by 20S proteasomes. Indeed, in certain in vitro experiments, the rate of proteolysis of non-ubiquitinated disordered proteins by 20S proteasomes was faster than by 26S proteasomes [49]. This could be explained by the hindrances to substrate processing and translocation found in the resting state of 26S proteasomes [39,40], nevertheless, it remains in question how disordered proteins can be targeted to 20S proteasomes and what activates these proteases for proteolysis. Apparently, certain features in disordered segments can engage with the 20S α-ring (Figure 2) and in this manner, some substrates can promote gating of the 20S proteasome to facilitate their own degradation [11]. Lacking ubiquitin-binding domains or ATPase subunits, 20S proteasome activity is adversely affected by the presence of globular domains within a partially unstructured polypeptide, or by conjugation of a substrate to the tightly folded ubiquitin. A recent study demonstrated that addition of ubiquitin units to a model disordered substrate, CyclinB1, slowed down the overall degradation rate of CyclinB1 by free 20S proteasomes [49]. Under such circumstances, 20S proteasomes either release the globular domain [11] or degrade the globular domain along with the unstructured portion of the substrate [49]. Despite its preference towards disordered proteins, detailed knowledge on how an unstructured polypeptide stretch is recognized as a signal by 20S proteasomes and what triggers a functional cycle is largely obscured. Further structural studies with substrate are necessary to address this issue.

## 3. Structural Precision of 20S Proteasomes for Substrate Degradation

**Figure 3:**
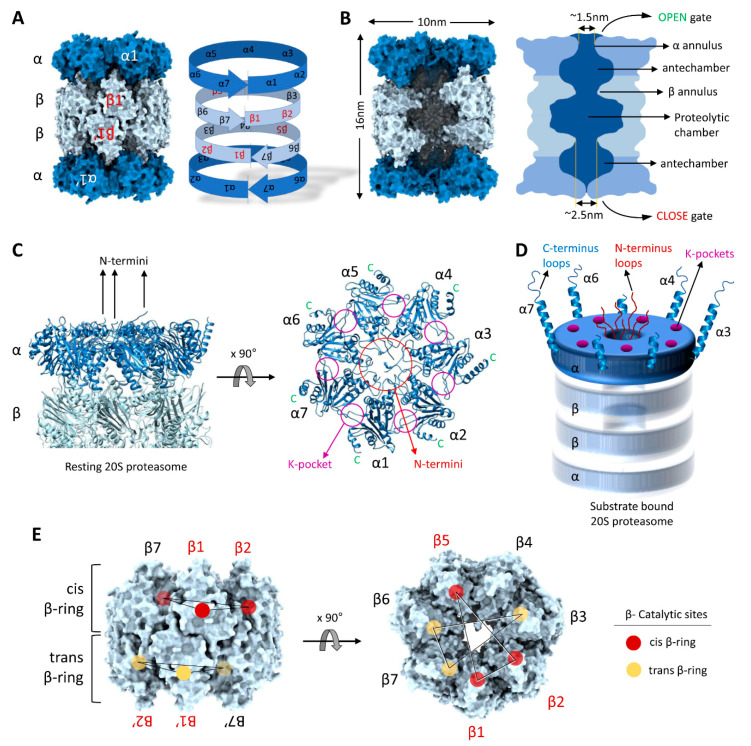
Structural precision of 20S proteasomes for substrate degradation. (**A**) The C2-symmetry of the 20S proteasome complex highlighting α-rings and the β-rings. (**B**) A slice through the interior of the 20S proteasome highlighting the proteolytic chamber separated from the two antechambers by β-annulus. (**C**) Central location of α-subunit N-termini and the peripheral positions of α-subunit C-termini in resting 20S proteasomes. (**D**) A cartoon representation of the N-termini, C-termini and K-pockets on the α-ring of substrate-engaged 20S proteasomes. (**E**) Spatial distribution of the β-subunit catalytic sites (β1, β2 and β5) in cis- and trans- β-rings. The model figures of 20S complexes were generated by ChimeraX using the 20S cryo-EM structure [49].

Structurally, the 20S proteasome is a hollow cylindrical barrel consisting of four rings—two peripheral α-rings and two central β-rings (Figure 2). Each α- or β-ring consists of seven homologous subunits (α1,2,3,4,5,6,7 or β1,2,3,4,5,6,7) positioned such that the C-terminus of each subunit faces outwards, while the N-terminus faces inwards. The upper and the lower αβ-rings (half-20S) are arranged in a reverse order: all four rings are aligned at α1/β1/β1/α1 subunits, with one half-20S running clockwise from subunit 1 to 7 whereas the other half-20S is arranged counterclockwise, providing an overall C2 symmetry to the 20S barrel (Figure 3A). The inner cavity of the 20S proteasome is divided into a central catalytic chamber lined by all the fourteen β-subunits, and two antechambers enclosed by the α-subunits (Figure 3B). Although the function of the β-catalytic subunits in the catalytic chamber are very well defined, the contribution of the two antechambers towards 20S proteasome function is not as clear [89,90,91]. An antechamber is not a strict requirement for a self-compartmentalized protease, since the proteasome-related prokaryotic HslV contains only two β-type rings enclosing a sole catalytic chamber [92]. 

Access to the inner cavity of the 20S proteasome is through a channel in the center of the α-rings. In the resting “latent” state, the channel is sealed by the intertwining extended N-termini of all seven α-subunits [20,22]. Upon binding of a regulator (19S/ PA200/ PA28), these N-termini shift, opening or closing depending on the proteasome functional state and hence this region is called the “gate” of the 20S CP [93,94,95,96]. However, even in absence of any regulator, 20S proteasomes can still degrade certain substrates—occasionally faster than 26S proteasomes—implying that the gate can also be opened by substrates with specific features [11,49,97]). At this time, it is unclear how substrates access free 20S proteasomes; however, structural features of the α-ring provide possible substrate-binding interfaces. For instance, in the substrate translocating state of 26S proteasomes, disorderedness is apparent in the extended N-termini of the α-subunits [98] implying that even in the 26S, the gate into the core particle is not merely “open” but rather the α-subunit N-termini retain the potential to interact with the substrate as it slides through. By analogy, substrates may interact directly with these N-termini in free 20S proteasomes, facilitating disorderedness and gate opening (Figure 3C).

C-terminal helixes at each of the seven α-subunits provide another potential medium for substrate association with 20S proteasomes. Each α-subunit terminates in an extended helix-loop motif projecting outward from the 20S proteasome surface (Figure 3D). The lengths of these C-termini vary from 15 to 40 aa residues, the longest of which (>30 aa) belong to α3, α4, α6 and α7. Since these termini are not completely resolved in Cryo-EM images or in crystal structures of 20S proteasomes, they are most likely flexible in nature. Such long flexible helix-loops provide putative binding interfaces for substrates with unstructured stretches. Reportedly, the C-terminus of the α7 subunit interacts with retinoblastoma (Rb) protein, p21, and Cdc25C proteins and facilitates their degradation in a ubiquitin-independent manner [83,99,100]. A third potential module for substrate interactions with 20S proteasomes are the lysine-pockets (K-pockets) on the outer α-surface (Figure 3C,D). Seven lysine-pockets are documented to bind a HbYX motif at the C-termini of most activators of 20S CPs, triggering gate opening [94,96,101,102,103,104,105]. Short peptides bearing such HbYX motifs at their C-terminus are reported for their ability to induce 20S CP gate opening, and so are some proteins or peptides that do not strictly adhere to the HbYX rule [106,107]. We propose that this mode of regulation could be utilized by substrate-proteins bearing similar motifs inserting into lysine-pockets on 20S CP surface to trigger gate opening and enhance their own degradation. Furthermore, 20S gating can be regulated by post-translation modification on the proteasome subunit itself. It has been reported that, under oxidative stress S-glutathionization on specific cysteine residues (Cys76 and C221) of the α5 subunit modulates 20S gate opening for proteolysis of oxidized proteins [86,87,108].

After entering the gated channel into the 20S proteasome, substrates first traverse an antechamber defined by the α-subunits and only then enter through a ~2.5nm diameter aperture defined by the β-annulus into the central proteolytic chamber (Figure 3B). The catalytic chamber is a central oval-shaped cavity about 5–6 nm wide. Notably, of all fourteen β subunits, only six subunits in eukaryotic 20S proteasomes have the active Threonine nucleophile at the N-terminus (by post-translational N-terminal trimming) [90,109]. The catalytic sites of the enzymatic β-subunits (β1, β2 and β5) face towards the center of the cavity (Figure 3E). Between them, they can cleave most peptide bonds since the β1 enzyme shows caspase-like (post acidic amino acid), β2 displays trypsin-like (post-basic), and β5 exhibits chymotrypsin-like (post-hydrophobic) specificities [89,110,111]. Since the two β-rings stack over the β1–β1′ pair and run in opposite directions to each other, the two other catalytic subunit pairs, β2–β2′ and β5–β5′, are not located one over the other (Figure 3E). The dispersed arrangement of few active subunits in the double β-ring (6 out of 14) leaves a gap at the β4 subunits where no in proteolytic sites are present. In contrast to primordial 20S proteasomes where all 14 β-subunits are catalytically active, it is possible that this “proteolytic gap” affords for partial cleavage of the substrates and generation of slightly longer peptide products that retain sequence information for downstream signaling pathways (see heading 5).

## 4. The Functional Cycle of 20S Proteasomes

20S proteasome structure has been studied in great detail at atomic resolution and extensive information has been amassed regarding its assembly, catalytic mechanism and modes of activation [24,112,113]. In this section we will concisely discuss substrate-induced conformational changes to 20S proteasome structures and attempt to ascribe hypothetical functional states during substrate degradation. Cross-referencing available biochemical and structural studies, we propose the following functional states: (1) substrate accepting state, (2) substrate binding state, and (3) substrate processing state.

### 4.1. Substrate Accepting State (SA)

Under most in vitro conditions, the 20S proteasome is found in a latent form with a closed gate conformation [20,79,102,104]. The N-terminal loops of the α-subunits extend into the center of the α-ring coming into close contact to form a plug with a few termini curving upwards at the epicenter (Figure 3C). The close gate hinders random entry of polypeptides, even of small tetra-peptides (such as the commonly used LLVY-AMC substrate), hence it is often referred to as the latent 20S CP. In its latent form, a free 20S proteasome is ready to accept potential substrates with loosely folded stretches; however, how these substrates are recognized by the 20S proteasome is largely obscure. As mentioned above, in the substrate accepting state, a few structural features on the α-rings may interact with potential substrates: (a) the flexible N-terminal loops that form the gate, (b) through the extended C-terminal loops, or (c) through the lysine-pockets (Figure 3D). These multiple modes of substrate association probably facilitate the gate opening and lead to further conformational changes that increase affinity of binding and engagement of the substrate.

### 4.2. Substrate Binding State (SB)

**Figure 4:**
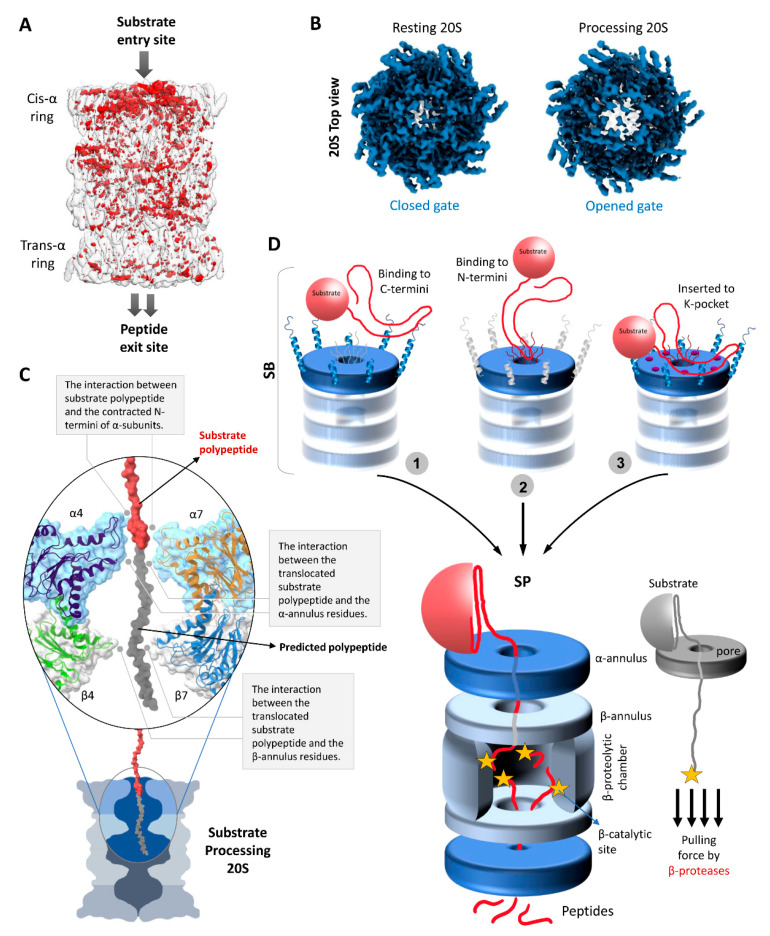
Structural conformational changes during substrate processing and functional model of the 20S proteasome. (**A**) A differential map rendering the asymmetric pattern between the trans- and cis- α-rings of substrate-processing human 20S proteasomes [49]. (**B**) The top view electron density map exposing the cis α-ring of resting 20S proteasome vs. substrate processing 20S proteasome [49]. (**C**) Potential interactions of a substrate polypeptide with the contracted N-termini of α-subunits, α-annulus residues, and β-annulus residues during substrate processing state. The surface and embedded cartoon representation of α4/7- and β4/7 along with the substrate polypeptide (in red) image was generated using the model of substrate-bound human 26S proteasomes (PDB: 6msk). A putative extension of the substrate polypeptide is colored grey for illustration purposes. (**D**) A model mechanism of substrate processing by 20S proteasomes. In substrate accepting state, we propose that the unstructured portion of the substrate would recognize and interact either with (1) the protruding C-termini of α-subunits, or with (2) the long N-terminal loops of α-subunits, or (3) may insert into K-pockets of α-subunits. The pulling force of β-proteases aids unfolding of the remainder of the substrate against the α- and β-annulus (aperture) by a ratchet mechanism.

The first direct evidence that substrate enters at the center of the α-ring was obtained by negative staining EM of stalled archaeal 20S proteasomes [78]. Interestingly, archaeal 20S proteasomes can engage the substrate at both sides of the complex simultaneously [78,114]. However, more recently, an unstructured substrate (CyclinB1) was shown to trigger gate opening in one of the α-rings of the free human 20S proteasome and induce conformational changes to half of the 20S barrel (Figure 4A,B) [49]. This result is in line with another recent demonstration that attachment of simple regulatory particles to archaeal 20S proteasomes introduced extensive allosteric changes extending from one outer α-ring to the catalytic sites at the center but not to the other half of the 20S barrel [115]. Furthermore, in cryo-EM images of substrate-engaged 26S proteasomes, the distal surface of the 20S CP remained closed [98,116]. Together, these observations suggest that human proteasomes can engage the substrate and trigger gate opening at one side, resulting in an asymmetric conformational change of the 20S barrel (Figure 4A).

Just below the gate into the proteolytic channel of the 20S proteasome there is a relatively stable narrow aperture lined by loops from all the α-subunits; the α-annulus. Interaction with these loops most likely provides substrate engagement to ensure entry into the antechamber (Figure 4C). Moreover, the aperture diameter (~1.5 nm) is compatible with a loosely folded polypeptide for interaction as it translocates through. A study by the Thomas and DeMartino groups reported that two extended polypeptides can cotranslocate through the same gate simultaneously; however, the fused globular GFP domain was released without getting degraded [11]. Further evidence that more than two polypeptides can enter simultaneously through the same gate and annulus stemmed from a recent study demonstrating that free 20S can proteolyse a Lysine48-linked tetraUb chain when attached to an unstructured substrate—CyclinB1 [49]. In the absence of any unfoldase/ATPase modulator, how the 20S proteasome upholds the ability to unfold and translocate a tightly folded protein such as ubiquitin remains to be elucidated. Given these biochemical observations, it would be interesting to investigate whether the 20S proteasome maintains these abilities in cells where it may associate with unfoldases/ATPase modulators (other than 19S), non ATPases activators or chaperons to aid degradation of small globular domains or proteins.

### 4.3. Substrate Processing State (SP)

After binding at the α-subunits, the substrate must translocate through the antechamber into the catalytic chamber for proteolysis. In order to do so, the substrate polypeptide has to enter through yet another aperture of the β-annulus. Though its diameter (~2 nm) is comparatively wider than α-annulus aperture, it might provide yet another interaction/contact point to the substrate polypeptide for translocation towards the catalytic chamber (Figure 4C). Entrapment by the β-subunit active sites provide a pulling force for continuous inward movement of the substrate polypeptide. A conceptual mechanism for “protease-like unfolding” by the group of Zhou has demonstrated that a polypeptide pulled through a narrow aperture in a wall leads to one-by-one breaking of hydrogen bonds, bypassing the initial force barrier required to overcome the tertiary structure of a globular protein [117]. Such a ratchet mechanism by a protease through a narrow aperture requires lower energy of unfolding than that of an energy-dependent ATPase. Specifically, pulling ubiquitin by its C-terminus against a nanoscale aperture yielded a mechanical force that unfolded the native conformation. Likewise, it is possible that ubiquitin conjugated at its C-terminus to an unfolded polypeptide long enough to reach the β-subunit protease sites can be unfolded by free 20S proteasomes as it is pulled through the α-ring aperture. In absence of any unfoldase, 20S proteasomes may follow a similar mechanism of substrate unfolding by a β-enzyme pulling action through two such narrow apertures (α- and β-annulus) for efficient proteolysis (Figure 4D). Attaching an antechamber to a catalytic chamber and requiring substrates to traverse a number of narrow apertures provides the contact points for 20S-directed proteolysis. Nevertheless, for the execution of substrate translocation sufficient evidence from structural data and the associated conformational changes are missing from our current understanding. Notably, the two different substrate translocation mechanisms by 26S and 20 proteasomes resulted in different peptide product outcomes despite the same catalytic core and potential endopeptidase cleavages by these two proteasome types [49].

## 5. Peptide Generation by the 20S Proteasome

Proteolysis of a substrate polypeptide inside the self-compartmentalized catalytic chamber of the 20S proteasome is highly regulated and modulated by the upstream regulatory mechanism. Under typical physiological conditions, both 20S and 26S proteasomes cleave protein substrates into small peptides ranging between 3 and 23 amino acids in length [118,119]. It is likely that proteasomes also liberate free amino acids, though fewer efforts have been made at documenting free amino acids among proteasome products. Mostly the proteolytic process is processive so that a protein is hydrolyzed within the catalytic chamber to the final products before the next substrate enters; hence, the pattern of peptides generated from a specific protein is relatively stable over time [119,120]. In the catalytic chamber of the 20S CP, each of the three catalytic β-subunits (β1, β2 and β5) preferentially cleaves after specific amino acids: β1 cleaves after acidic or small hydrophobic amino acids, β2 cuts after basic or small hydrophobic amino acids, while β5 hydrolyzes the peptide bond after hydrophobic residues whether bulky or not [110]. However, the rules that govern the cleavage rate and specificity of the same peptide bond can be significantly altered upon attachment of a 19S regulatory particle. An interesting feature of proteasome-dependent proteolysis is that the 20S and the 26S proteasomes generate different patterns of cleavage products [121], indicating that the distal 19S RP affects the behavior of the 20S CP when put into the context of the 26S proteasome. Attachment of proteasome activators not only influences substrate selection, but may also affect product outcome due to allosteric effects on β-catalytic active sites [118]. For example, both in vitro and in vivo conditions, 20S and 26S proteasomes generate different peptide products from an identical substrate protein with respect to their types, amount and sizes [49,118,121,122]. Interestingly, it has been shown that cells with high 20S proteasome levels, as occurs under hypoxia or human cardiomyopathies, generate elevated amounts of ubiquitin-derived peptides [49], suggesting that some of the ubiquitin tag is proteolyzed along with the conjugated substrate. Similar to 19S RP, the presence of alternative caps (PA28 or PA200) on the 20S CP affect the product outcome from the same substrates [123]. Furthermore, the CP contains other “noncatalytic” sites to which additional factors can bind and alter cleavage sites or product composition [124].

It has been reported that free 20S proteasomes generate longer peptides than do 26S proteasomes [49,118,121] suggesting the gating mechanism differs in both proteasome species. The majority of peptides generated by the 26S proteasome contain less than eight amino acids. A fraction of the peptides that are 8–10 amino acids in length can be transported through the ER and presented to the immune system by MHC class I [125,126]. An increase in average peptide product length by shifting proteasome population from 26S to 20S proteasomes could increase the efficiency of antigen presentation and, by extension, the efficiency of combating viral infection. Understanding the precise rules regulating the makeup of peptides generated by different proteasome species could have far-reaching consequences on predicting immunogenic peptides “hidden” within viral or tumorigenic proteins. Generally, the peptide products are short lived and most of these peptides are likely to be rapidly hydrolyzed by downstream proteases and aminopeptidases. However, some peptides persist in the intracellular peptide pool [127,128]. Apart from the known immunogenic function, these intracellular peptides generated by proteasomes may have the potential to modulate other prospective signaling pathways [129]. Hence, the diverse peptide products from different proteasome species would have high propensity to modulate cellular signaling pathways under various physiological conditions; this, however, needs further investigation.

## 6. Concluding Remarks

Proteasomes are the major intracellular proteases for regulated protein degradation. Most cellular proteins can end up as proteasome substrates either in a regulated manner upon specific molecular cues, or nonspecifically if damaged or misfolded. Finding both the tightly regulated 26S proteasome and the simpler free 20S complex side-by-side in most eukaryotic cells implies that each may have independent roles in protein homeostasis. Although the 20S complex is a less regulated proteasome than the 26S holoenzyme and limited to unstructured polypeptides, its levels increase under various human pathologies, suggesting that a proper proteasome ratio may be important for overall protein homeostasis. Overall, 20S proteasomes appear to play an emergency role contributing survival benefits to cells under physiological stress.

The signals that target substrates for degradation are distinct for 26S and 20S proteasomes. While ubiquitination is the major criterion for substrate targeting to the 26S, an unstructured stretch is required for substrate proteolysis by the 20S proteasome. In principle, an unstructured stretch could engage at both proteasomes; however, specific ubiquitin-triggered conformational changes render 26S proteasomes particularly appropriate for ubiquitin-dependent degradation. Likewise, unstructured polypeptides may induce conformational changes to 20S proteasomes for their own degradation. By implication, the substrate repertoire of the two proteasomes is not fully overlapping, which is intriguing since the two proteases have identical catalytic active sites in their cores.

Precise knowledge of how 20S proteasomes work as molecular machines is essential to clarify how they prioritize their substrates in the cell. The current knowledge of 20S proteasomes’ structural features provides partial information about how these complexes recognize substrates and process them. However, further dissection of 20S proteasome mechanisms awaits single-particle analysis with its appropriate substrates, which will unravel the 20S proteasome in action. Additionally, describing the regulatory role of auxiliary factors, proteasome-interacting proteins, post-translational modifications, and cellular and physiological conditions will complete our understanding of 20S proteasome function. Developing approaches to study proteasomes in living cells will clarify how different proteasome species (20S and 26S) contribute to overall intracellular proteolysis. The resulting information will aid strategies for the targeted inhibition or modulation of proteasome activities.
